# Anxiety, depression, and academic stress among medical students during the COVID-19 pandemic

**DOI:** 10.3389/fpsyg.2022.1066673

**Published:** 2023-01-11

**Authors:** Lorena Avila-Carrasco, Daisy Lorena Díaz-Avila, Adrian Reyes-López, Joel Monarrez-Espino, Idalia Garza-Veloz, Perla Velasco-Elizondo, Sodel Vázquez-Reyes, Alejandro Mauricio-González, Jorge Alfonso Solís-Galván, Margarita L. Martinez-Fierro

**Affiliations:** ^1^Academic Unit of Human Medicine and Health Sciences, Zacatecas Autonomous University, Zacatecas, Mexico; ^2^Pathology and Molecular Diagnosis Laboratory, Academic Unit of Chemical Sciences, Zacatecas Autonomous University, Zacatecas, Mexico; ^3^Molecular Medicine Laboratory, Academic Unit of Human Medicine and Health Sciences, Zacatecas Autonomous University, Zacatecas, Mexico; ^4^Academic Unit of Electric Engineering, Zacatecas Autonomous University, Zacatecas, Mexico

**Keywords:** academic stress, anxiety, COVID-19, depression, medical students

## Abstract

**Background:**

The social distancing policies implemented by the health authorities during the COVID-19 pandemic in Mexico and elsewhere led to major changes in teaching strategies for college undergraduates. So far, there is limited data regarding the impact of the lockdown on the academic stress and mental health of these students.

**Objective:**

To assess the occurrence of academic difficulties, anxiety, depression, and academic stressors resulting in somatization with subsequent coping strategies linked to the pandemic.

**Materials and methods:**

A cross-sectional study was conducted with 728 medical students (years 1–5). A purposely designed questionnaire to assess academic difficulties associated with the pandemic was administered electronically. The validated Goldberg anxiety and depression scale was also used, as well as the SISCO-II inventory on academic stress.

**Results:**

Screening for anxiety and depression led to a prevalence of 67.9 and 81.3%, respectively. Most relevant stressors, reported always or nearly always, included professors’ evaluations (63.9%), and reading overload of academic papers (50.6%). Factorial analyses showed that women were more prone to stress than men (*p* < 0.001). Somatization symptomatology included drowsiness or increased need of sleep, anxiety, anguish, desperation, chronic fatigue, and sleep disorders. Common coping strategies included practicing a hobby, done always or nearly always by 65% of students with high stress, and 34% of those with low stress (*p* < 0.001).

**Conclusion:**

There was a relevant impact of the mandatory lockdown during COVID-19 pandemic on the mental health of medical students reflected in the high prevalence rates of anxiety, depression, and stressors in the studied population pointing to the need for designing and implementing preventive strategies to deal with the effects of lockdowns.

## 1. Introduction

Coronavirus 2019 disease (COVID-19) is a viral infection that can lead to the potentially lethal severe acute respiratory syndrome coronavirus 2 (SARS-CoV-2). It appeared in December 2019 in Wuhan, Hubei province of China, and since then it spread rapidly throughout the world (Hopkins, 2020; Yang and Wang, 2020; World Health Organization, 2022).

As to December 2022, the pandemic has caused more than 6.62 million deaths, and it has had a major economic impact coupled with a transformation of the daily life everywhere (World Health Organization, 2022). The social isolation that arose from the mandatory lockdown implemented by many countries has resulted in various psychological problems for the population (Wang et al., 2020). Recent reports show that COVID-19 lockdowns, including curfews, quarantines, and other societal restriction measures, are associated with symptoms of acute stress disorder (ASD) and posttraumatic stress disorder (PTSD), along with other issues such as emotional exhaustion, lack of interest, and mental disturbances including anxiety and depression (Brooks et al., 2020; Nelson et al., 2020).

Among students of medicine and other academic tracks, stress (i.e., the body’s reaction to physical, mental, or emotional pressure) is an important concern (Behere et al., 2011), whereas the events responsible for its occurrence are called stressors. Yet, people can react differently to the same stressor; the response can be physical, emotional, or behavioral, and it is usually framed within the physical and social environment of the individual. Such response appears when the assessment of an event appears as a threat to the individual’s wellbeing, and especially when he/she lacks the resources to face it and cope with it (Arribas Marín, 2013; Cohen et al., 2016).

Stress affects the lives of many people’s around the world, and it is linked to the psychopathology of various health conditions, and to their inability to adapt to challenging social environments (Eisenbarth et al., 2019). Stressful life experiences and the individuals’ psychological condition influence the clinical manifestations and course of many diseases (Straub and Cutolo, 2018). Back in 1948, Selye theorized that a stressor generally produces an acute response (alarm reaction stage) that can be adaptive (stage of resistance) and resolve or lead to an exhaustion stage with potentially chronic and irreversible physiological, psychological, and psychosocial consequences (Selye, 1948). A theoretical framework on the response to stress (i.e., allostasis) states that the allostatic load could accumulate resulting in an overexposure to stress mediators (immunologic, endocrine, and neural) triggering adverse organic effects that can lead to illnesses (McEwen, 1998). The theory of cognitive activation of stress is centered on the coping response that seems to determine the outcome, potentially resulting in stress-related illnesses if coping strategies are insufficient or inadequate (Ursin and Eriksen, 2010). Continuous thinking about negative events, passed or future (i.e., perseverative cognition), manifested through worry, rumination and brooding, has also been linked to stress-related problems, including cardiovascular, endocrine, and immunologic (Brosschot et al., 2006; Segerstrom et al., 2008).

Additionally, many university students experience academic stress associated with educational activities (e.g., exams, assessments, study load, etc.) and financial difficulties that have an impact on their learning performance (Crego et al., 2016). Students can also develop psychophysiological manifestations including memory and concentration difficulties, mental blocks, chronic fatigue, drowsiness, and despair (Barraza Macías, 2008).

Medical students are particularly prone to high levels of stress and psychological disorders such as anxiety and depression (Guthrie et al., 1995; Toews et al., 1997; Moffat et al., 2004). Studies have shown that most medical students experience different forms of psychological distress (Dyrbye et al., 2005, 2006; Heinen et al., 2017; Zvauya et al., 2017), some of which can lead to a higher risk of suicidal ideation and thoughts about quitting medical studies (Dyrbye et al., 2011). Students under stress try to deal with stressors by means of physical, psychological, or social behavioral responses; these coping strategies attempt to overcome or at least attenuate stress situations.

The social distancing policies implemented by the government and health authorities during the COVID-19 pandemic in Mexico and many other countries led to major changes in teaching strategies, including those for medical students, with internet-based learning becoming the cornerstone of these efforts for more than a year. However, so far there is limited data on what has been the impact of the lockdown on the mental health and academic stress in these medical students. Therefore, this study was undertaken to assess the occurrence of academic difficulties, anxiety, depression, and academic stressors that resulted in somatization and coping strategies linked to the pandemic in medical students from a Mexican university. The study proposal was reviewed and approved by the Ethics and Research Committee at the Academic Unit of Human Medicine and Health Sciences of the Autonomous University of Zacatecas (approval ID: AMC/CI/02-ORD/2020, CEICCL0121- ALGDEZA-03). All participant students provided signed informed consent.

## 2. Materials and methods

### 2.1. Study design and participants

This was a cross-sectional study conducted with 728 medical students out of the 928 registered at the Autonomous University of Zacatecas, in the homonymous State in the Central Northern region of Mexico. Male and female students from years 1 to 5 were surveyed. At the time of the survey (early Spring 2021), internet-based learning was the educational modality due to the compulsory lockdown.

### 2.2. Measuring instruments

#### 2.2.1. General questionnaire

This was a purposely designed tool that contained basic sociodemographic data and a number of questions assessing academic difficulties potentially linked to the COVID-19 pandemic.

#### 2.2.2. Goldberg anxiety and depression scale (GADS)

This screening tool consists of two subscales, one for anxiety and the other for depression, each with nine items with dichotomous responses (Goldberg et al., 1988). An independent score is totalized for each scale. Questions 1–9 relate to anxiety (e.g., Have you had difficulty relaxing?), and questions 10–19 relate to depression (e.g., Have you felt low energy?); the first four questions in each subscale are conditioning questions, so that two and one positive answers are needed to continue answering each subscale, respectively. The individual is questioned about whether he/she has presented any of the relevant symptoms; those lasting <2 weeks or are of mild intensity are not scored. The cut-off points for anxiety and depression used in this study was ≥4 and ≥ 2, respectively. An adequate internal and external validity has been reported; correlation coefficient with the Hamilton Depression Scales is 0.74 (Holm et al., 2001). A total of 728 students completed this questionnaire.

The validity and reliability of the Goldberg anxiety and depression scale has been assessed in two Spanish speaking Latin American countries; one with a sample of 548 Cuban adults that showed a good diagnostic performance (AUC: anxiety 0.64, depression 0.68) (Martín-Carbonell et al., 2016), and another with a sample of 609 psychology students from the Catholic University of Cuenca, Ecuador, that showed a good reliability (Cronbach’s alpha: anxiety 0.75, depression 0.80) (Reivan-Ortiz et al., 2019). In research studies, GADS has been previously used to assess depression symptoms among middle-aged Latin American women with severe menopausal symptoms and obesity (Blümel et al., 2016), to assess anxiety and depression among Mexican patients with dystrophic epidermolysis bullosa (Fortuna et al., 2016), and more recently in two Mexican studies, one with type 2 diabetes patients (Gonzalez Heredia et al., 2021), and another with primary caregivers of patients in dialysis (Gonzalez Heredia et al., 2021).

#### 2.2.3. SISCO-II psychometric inventory on academic stress

This instrument contains eight questions that assess academic stressors (e.g., peer competition, homework overload, teachers’ personality, etc.), and another 17 items that measure physical, psychological, and social behavior reactions (e.g., sleep disorders, chronic fatigue, headache, etc.) in response to academic stress (i.e., dimensions of somatization). An additional six questions measure coping strategies (e.g., ability to defend preferences without harming others, religious practice, information seeking about a situation, etc.) with five possible answers each using a Likert scale (never, seldom, sometimes, nearly always, always) were part of the instrument.

The SISCO-II inventory of academic stress has been considered valid and reliable scale for diagnostic use. It was designed and tested in Mexico showing good reliability (Cronbach’s alpha: stressors 0.85, symptomatology 0.91, and coping 0.69) (Barraza, 2007). It has been validated in other Latin American countries such as Colombia (Malo et al., 2010), Perú (Manrique-Millones et al., 2020), and considered appropriate in these three countries. Thus, it has been considered a valid tool for use in Latin American students (Castillo-Navarrete et al., 2020). A total of 421 students completed this questionnaire.

### 2.3. Data collection

Students were sent a link where they could access the webpage to participate in the survey, which lasted between 15 and 20 min. Students were sent a reminder email one week after the first contact. All questionnaires were administered electronically using “Google forms.” Participants had to provide informed consent in the first page of the survey to be able to continue. Data was collected anonymously in March 2021. Overall participation rate was 76.1% (728/956), but for the SISCO-II inventory it was 44% (421/956). No data were available for the reasons for not responding.

### 2.4. Statistical analysis

Percentages were used to describe issues and problems associated with the online teaching modality during the pandemic. Overall prevalence of anxiety and depression was also computed with results stratified by sex an age. Density analysis was used to graph the most frequent responses. An exploratory analysis relating anxiety and depression was done, as these psychiatric illnesses are known to be highly comorbid with each other (Kalin, 2020).

Percentages were used to present the response level for the SISCO-II inventory. Clusters were produced to stratify the population based on the stressors assessed. Responses obtained for each stressor were assessed by K-means. Also, an exploratory factorial analysis was used to reduce the number of variables included in the instrument using various methods (e.g., Bentler, acceleration factor, R^2^, VSS complexity 1, Velicer’s MAP, TLI, RMSEA, optimal coordinates, parallel analysis, and Kaiser criterion); factors were used to assess whether there were significant differences between clusters based on symptomatology. Correlations between stressors were done using Kendall rank correlation coefficients; item dependency was considered statistically significant with *p* values below 0.05.

## 3. Results

From the 728 surveyed medical students from years 1 to 5, 301 (41.3%) were men and 427 women (58.7%). Most students (96%) were aged 18–24 years; differences in sex for grade was not significant (χ2 = 3.24, value of *p* = 0.52; Table 1).

**Table 1:** Frequency distributions by sex and age according to grade level.

Degree course	*n*	Male	Female	Age (mean ± SD)
1	122	49	73	18.72 ± 0.92
2	106	43	63	20.13 ± 1.94
3	142	52	90	20.93 ± 0.92
4	172	71	101	21.97 ± 1.01
5	186	86	100	23.11 ± 1.33

### 3.1. Educational difficulties reported during COVID-19 pandemic

**Figure 1:**
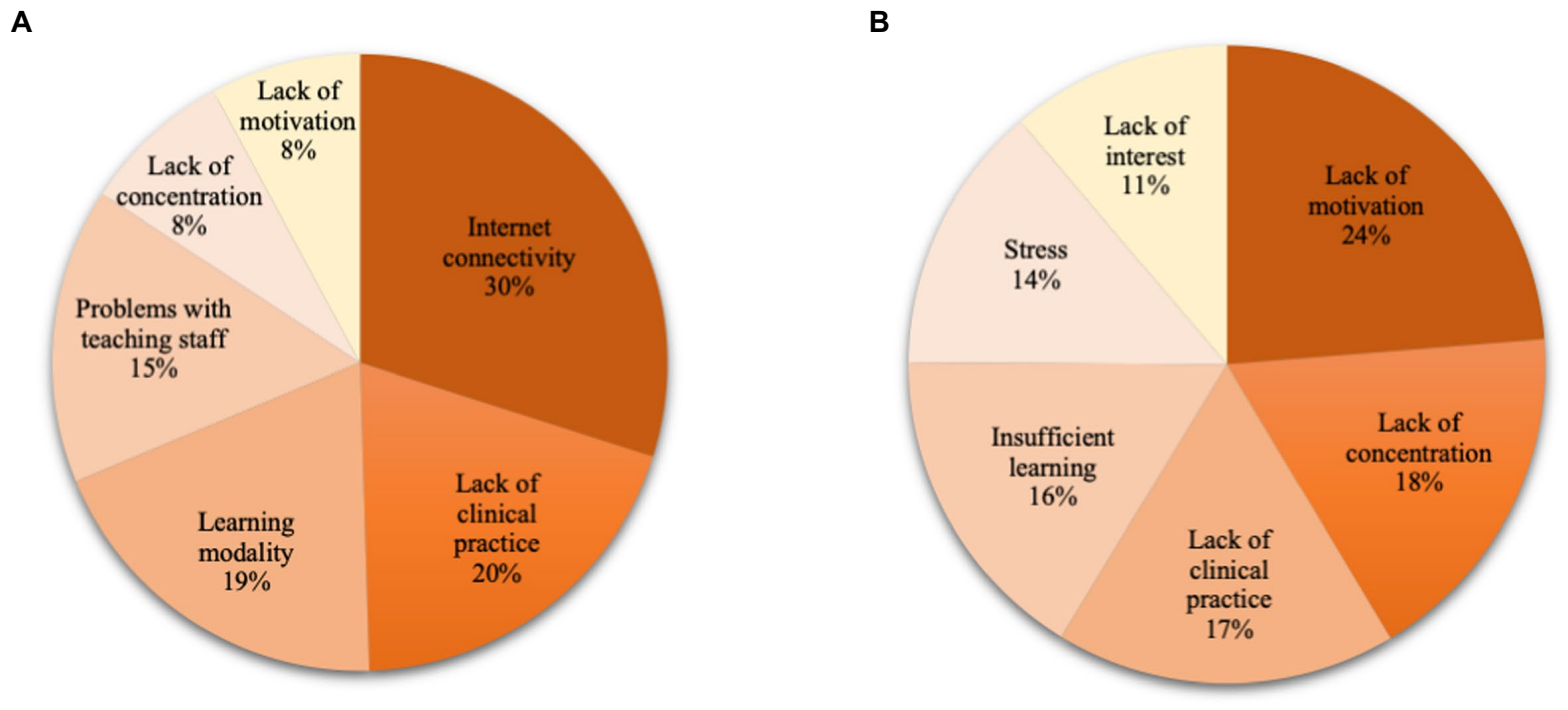
Main educational problems associated with the COVID-19 pandemic: (**A)** problems with learning modality and (**B)** resultant academic problem.

The main educational difficulties referred to by the students are presented in Figure 1A. The most common difficulty associated with the learning modality was “problems with internet connectivity,” reported by 30% of the students, followed by the “lack of clinical practice (hospital and/or laboratory)” with 20%. Students also reported “problems with the internet learning modality” (19%) and “problems with the faculty” (15%). “Lack of concentration and motivation” was also reported by 8% of the students each. As to students’ academic performance in the pandemic, 92% reported being affected. Most frequent reasons given were “lack of motivation” (25.2%), “lack of concentration” (18.8%), “lack of clinical practice” (18.4%), “insufficient learning” (17.2%), and “stress” (14.6%; Figure 1B).

### 3.2. Prevalence of anxiety and depression

Overall, screening for anxiety and depression led to a prevalence of 67.9 and 81.3%, respectively. Women had significantly higher proportions of both, anxiety (73.3% vs. 60.4%; *p* < 0.001) and depression (87.1% vs. 73%; *p* < 0.001), but no statistical differences were seen by age (Table 2). However, those aged 19 years or less showed a notably lower prevalence of anxiety (nearly 10 percent points below the other ages), and yet a higher prevalence of depression (6–12 percent points above the other ages).

**Table 2:** Prevalence of anxiety and depression according to the Goldberg scale by sex and age among medical students at the Autonomous University of Zacatecas, 2021.

Variable	Category	Frequency (%)					
		Anxiety^1^			Depression^2^		
		Negative	*p*-value*	Positive	Negative	*p*-value*	Positive
Sex	Male	119 (39.5)	<0.001	182 (60.4)	81 (26.9)	<0.001	220 (73.0)
	Female	114 (26.6)		313 (73.3)	55 (12.8)		372 (87.1)
Age (years)	≤19	33 (23.9)	0.17	105 (76.0)	16 (11.5)	0.10	122 (88.4)
	20	39 (34.8)		73 (65.1)	21 (18.7)		91 (81.2)
	21	53 (37.0)		90 (62.9)	30 (20.9)		113 (79.0)
	22	53 (33.3)		106 (66.6)	28 (17.6)		131 (82.3)
	≥23	55 (31.2)		121 (68.7)	41 (23.2)		135 (76.7)
Total	728	233 (32.0)		495 (67.9)	136 (18.6)		592 (81.3)

^1^Goldberg scale: ≥4 relevant symptoms (those lasting <2 weeks or of mild intensity did not score). ^2^Goldberg scale: ≥2 relevant symptoms (those lasting <2 weeks or of mild intensity did not score). *Pearson Chi-square tests were used.

**Figure 2:**
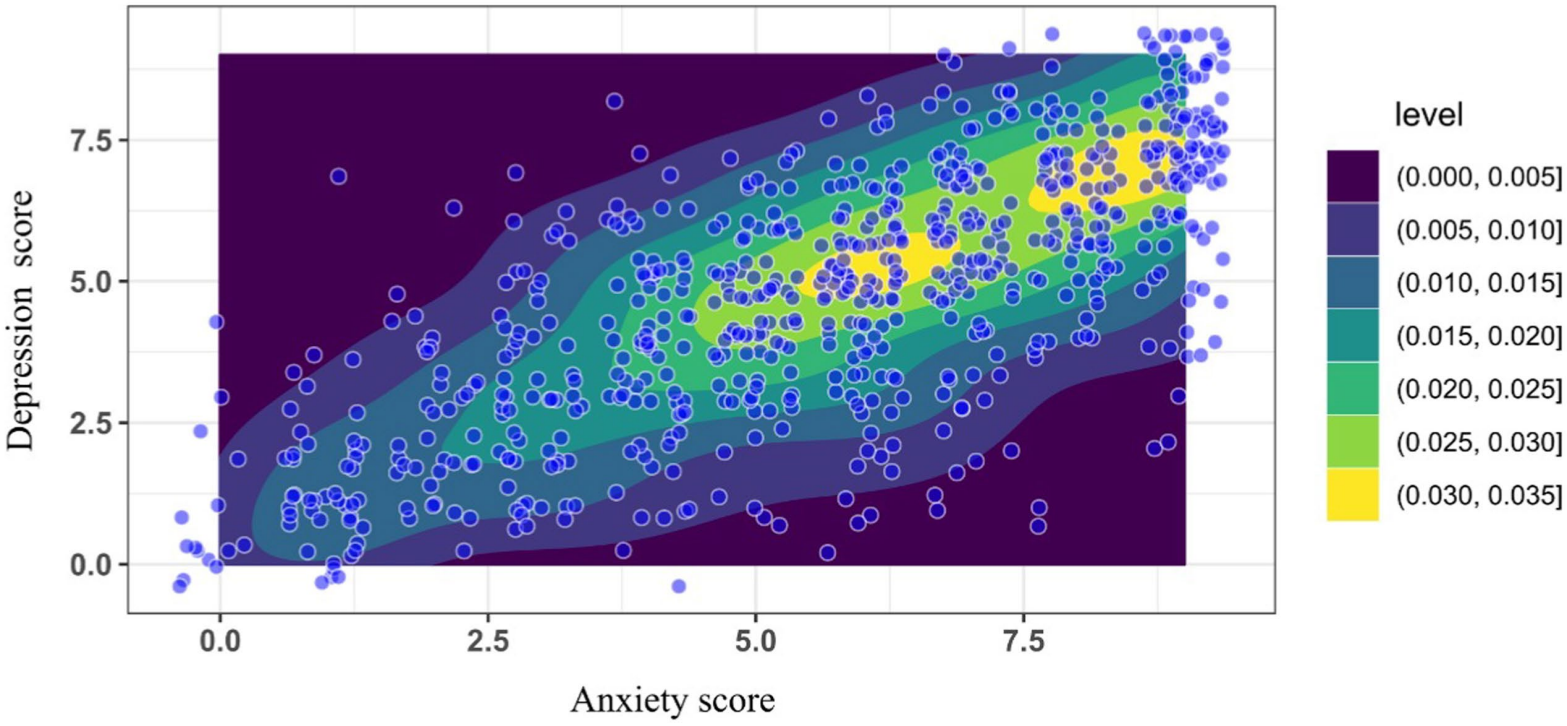
Linear regresión for anxiety as predictor of depresion.

Density analysis and linear regression showed that most frequent responses for anxiety were between 4 and 9, and 4 to 8 for depression. Linear regression between both domains (anxiety as predictor of depression) showed a Pearson coefficient of 0.529 (*p* < 0.05; Figure 2).

### 3.3. SISCO-II inventory on academic stress

The most frequent stressor was the “evaluation of professors” where 40.6% of students reported that caused then stress nearly always, and 23.3% as always, followed by “overload of tasks and academic papers” with 38 and 12.6%, and by “participation in class” with 26.6 and 20.9%, respectively. In contrast, “type of work that professors request,” and “competition with others” were items that caused less stress (Table 3).

**Table 3:** Distribution of stressors by response level among medical students (*n* = 728).

Stressors	Response level* (%)				
	1	2	3	4	5
Overload of tasks and academic papers	0.7	4.0	44.7	38.0	12.6
Personality and character of professors	2.6	12.6	43.9	32.1	8.8
Evaluation of professors (exams, tests, research, etc.)	0.5	6.4	29.2	40.6	23.3
Type of work that professors request	3.3	28.0	41.8	20.2	6.7
Do not understand topics covered in class	2.1	15.2	41.8	29.0	11.9
Participation in class (e.g., response to questions)	3.3	17.8	31.4	26.6	20.9
Limited time to do work	4.0	22.3	35.2	25.9	12.6
Competition with others in the group	9.3	24.7	35.9	19.2	10.9

*1 = Never, 2 = Seldom, 3 = Sometimes, 4 = Nearly always, and 5 = Always.

**Figure 3:**
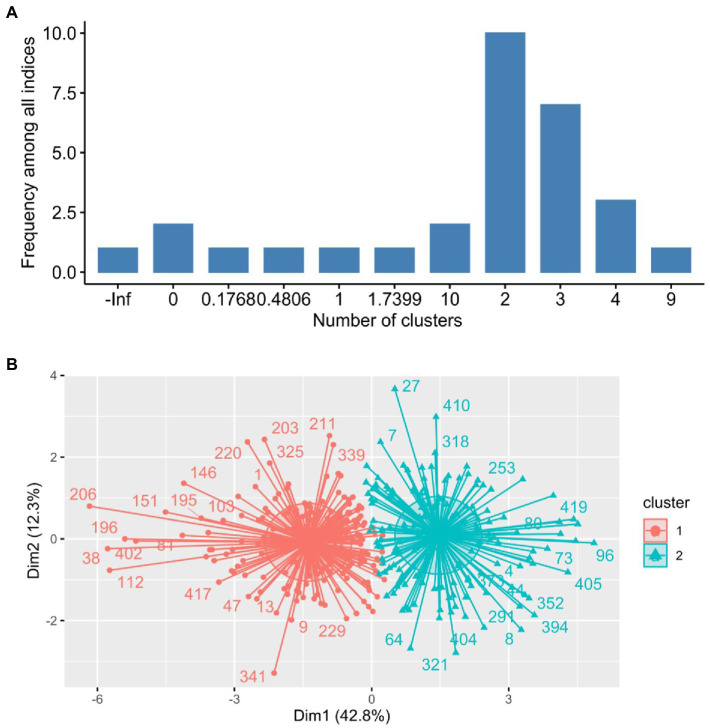
**(A)** Number of clusters to classify students according to stressors. **(B)** Cluster plot depicting the two groups (low and high stress).

The optimization of the number of groups to classify students according to the stressors resulted in two clusters (Figure 3A). The cluster plot produced two separated groups, one with low stress (*n* = 216), and another with high stress (*n* = 205; Figure 3B).

Women were more prone to stress than men (*p* = 0.0003), but no statistical differences were observed by mean age (Table 4).

**Table 4:** Differences in academic stress (low vs. high) by sex and mean age in medical students at Zacatecas Autonomous University, 2021.

Variable	Category	Low stress1	*p*-value*	High stress^1^
Sex	Male, *n* (%)	100 (62.8)	0.0003	59 (37.2)
	Female, *n* (%)	116 (44.2)		146 (55.8)
Age	Mean	21.5	0.20	21.3

^1^Grouping cut-off was based on the cluster analysis. *Pearson Chi-square test was use for nominal data, and student *t*-test for continuous data.

### 3.4. Dimensions of somatization

The distribution of somatization variables (i.e., physical, psychological, and social behavior response) by response level in groups 1 (low stress) and 2 (high stress) is shown in Table 5. The proportion of responses with levels 4 (nearly always) and 5 (always) were notably higher in group 2 (high stress) compared with group 1 (low stress), especially for “drowsiness or increased need for sleep,” “anxiety, anguish, or desperation,” “chronic fatigue (permanent fatigue),” and “sleep disorders (insomnia or nightmares).”

**Table 5:** Distribution of somatization variables by response level by stress groups.

Somatization variables	Response level* (%)				
	1	2	3	4	5
**Group 1 (low stress)** ^**1**^					
Sleep disorders (insomnia or nightmares)	14	29	27	24	6
Chronic fatigue (permanent fatigue)	6	24	37	26	6
Headaches or migraines	14	33	39	13	1
Digestive problems, abdominal pain, diarrhea	17	31	33	14	6
Scratching, nail biting, rubbing, etc.	19	25	23	22	11
Drowsiness or increased need for sleep	4	13	44	30	9
Muscle pain and/or contractures	21	28	30	16	5
Skin reactions (rash, peeling, etc.)	54	23	12	8	3
Restlessness (inability to relax and be calm)	8	25	38	24	5
Anxiety, anguish, or desperation	5	22	44	22	7
Increased or decreased food consumption	13	21	41	20	5
Feelings of depression and sadness (depressed)	9	24	43	20	4
Feelings of aggressiveness or increased irritability	12	29	38	18	4
Abrupt mood changes	19	29	30	17	4
Conflict or tendency to argue or debate	19	36	31	12	2
Isolation from others	17	28	38	15	3
Unwillingness to perform student duties	3	17	50	23	7
**Group 2 (high stress)** ^%**1**^					
Sleep disorders (insomnia or nightmares)	3	14	26	27	30
Chronic fatigue (permanent fatigue)	1	7	24	36	31
Headaches or migraines	4	18	38	26	14
Digestive problems, abdominal pain, diarrhea	6	22	33	22	17
Scratching, nail biting, rubbing, etc.	13	15	21	25	26
Drowsiness or increased need for sleep	0	5	25	36	34
Muscle pain and/or contractures	7	18	32	29	15
Skin reactions (rash, peeling, etc.)	35	26	21	8	10
Restlessness (inability to relax and be calm)	1	4	31	37	26
Anxiety, anguish, or desperation	1	5	28	32	34
Increased or decreased food consumption	4	12	28	32	24
Feelings of depression and sadness (depressed)	3	4	34	32	26
Feelings of aggressiveness or increased irritability	2	19	35	26	18
Abrupt mood changes	3	21	34	26	16
Conflict or tendency to argue or debate	10	28	34	14	14
Isolation from others	5	19	33	27	16
Unwillingness to perform student duties	1	3	29	37	30

^1^Grouping cut-off was based on the cluster analysis. *1 = Never, 2 = Seldom, 3 = Sometimes, 4 = Nearly always, and 5 = Always.

According to sex, the distribution of response level in somatization, we found significant differences in most of the items (*p* < 0.01), except for the cratching, nail biting, rubbing, etc. and Unwillingness to perform student duties items.

### 3.5. Coping strategies

Table 6 compares the response level of comping strategies by stress group. Most frequent coping strategies include “practicing a hobby,” performed nearly always or always by 65% of students with high stress and by 34% of those with low stress (*p* < 0.001), followed “getting company by a loved one” with 56 and 34% (*p* < 0.001), respectively. No statistical differences in responses between groups 1 and 2 were seen for strategies such as “ventilation and confidences,” “complimenting oneself,” and “assertive ability.”

**Table 6:** Comparison of coping strategies by response level between the two stress groups among medical students (*n* = 728).

Strategies	Group^1^	Response level* (%)				
		1	2	3	4	5
Developing of a plan to perform tasks	1	8	27	42	15	8
	2	8	15	43	21	13
Complimenting oneself	1	16	36	31	13	4
	2	20	37	29	8	6
Ventilation and confidences (e.g., verbalization of concerned situation)	1	11	40	35	11	4
	2	11	38	33	12	6
Assertive ability (i.e., defend preferences or feelings without harming others)	1	5	24	34	29	9
	2	5	29	38	20	7
Practicing a hobby (e.g., exercise, reading, TV, social networking, etc.)	1	2	10	24	45	20
	2	3	23	40	21	13
Getting company by a loved one (e.g., family, friends, pets, etc.)	1	3	15	26	30	26
	2	6	23	37	17	17

^1^Grouping cut-off was based on the cluster analysis: 1 = low stress, 2 = high stress. *1 = Never, 2 = Seldom, 3 = Sometimes, 4 = Nearly always, 5 = Always.

According to sex, the distribution of the level of response in the strategies, we did not find significant differences in any of the items.

## 4. Discussion

This study was carried out to assess the presence of academic difficulties, anxiety and depression, and academic stressors that result in somatization and coping strategies linked to the COVID-19 pandemic in medical students in a Mexican university.

Stress, in general, is a complex multifactorial process that can result in somatic symptomatology, including gastrointestinal problems, insomnia, insulin resistance and overweight/obesity, as well as adaptative disorders, memory alterations, and depression, among others (Tafet and Nemeroff, 2016). When stress occurs in an educational context, it is known as academic stress (Putwain, 2007), which is characterized by physiological, emotional, cognitive, and behavioral stimuli that happens as consequence of a student’s inability to cope with difficulties in a school setting; stressors linked to school procedures can also trigger this reaction (Barraza Macías, 2008). Academic stress associated with poor coping strategies may affect academic performance, school homework, exams, and poor learning (Eva et al., 2015). Therefore, timely identification of academic stressors is essential to prevent somatization and the development of more serious physical and/or mental illnesses (Zhai and Du, 2020).

Academic stress has been widely linked to several negative effects on academic performance, school homework, exams, and learning.

Nearly one out of three students reported difficulties with internet connectivity as the main problem linked to the learning modality, and one in five said that the lack of clinical practice was the main concern. Lack of motivation (24%), concentration (18%), and clinical practice (17%) were the most common academic performance concerns.

Research has shown disproportionally higher levels of stress, anxiety, and depression among medical students compared to nonmedical undergraduates (Alzahrani et al., 2020). The incidence of psychological dysregulation in medical students is often attributed to the competitive and stressful environment along with other academic and professional demands which often lead to emotional exhaustion and psychological distress (Abdel Wahed and Hassan, 2017).

Screening for anxiety and depression resulted in an overall prevalence of 68 and 81%, respectively. Results also showed sex differences for both anxiety and depression with higher proportions in women. These results come from data collected in March 2021, right after the second wave of the pandemic, when mortality was at its highest levels. During this period, strict social distancing measures were in place, leading to complete online teaching in all educational system. However, it is important to consider that even before the COVID-19 pandemic, medical students already had relatively high levels of stress and depression (Dyrbye et al., 2006). A meta-analysis from 2016 had revealed a global prevalence of depression of 28% with first year medical students having the highest rate (Puthran et al., 2016), and with estimates ranging from 8% in American universities assessed with the Center For epidemiologic Studies-Depression scale (CES-D) to as high as 70% in medical schools of Pakistan using the Aga Khan University Anxiety and Depression Scale.

Whether the COVID-19 pandemic has resulted in higher incidence of anxiety and depression is still a contentious topic. For instance, a recent review concluded that the overall prevalence of anxiety in medical students did not increase with the outbreak, as high levels of resilience and healthy coping systems were put in place along with a reduction of academic load together with available family support (Lasheras et al., 2020). Conversely, another study reported that most medical students felt significantly more distressed about their studies due to COVID-19 (Loda et al., 2020). Previous research has shown that stress in preclinical medical students varies depending on the setting, ranging from 20.9% to more than 90% (Fares et al., 2016). In Mexico, before the pandemic, there is data associating high levels of academic stress in medical students with various psychological outcomes such as mental blocks, despair, feelings of depression and anxiety, restlessness, concentration and memory difficulties, and other physiological manifestations including chronic fatigue, drowsiness, headaches, insomnia, and muscle tremors (Pozos-Radillo et al., 2016).

Most relevant stressors, reported as always/nearly always, included professors’ evaluations (63.9%), and reading overload of academic papers (50.6%). Other stressors previously reported include poor academic performance (Zeppa and Linn, 1984), excessive academic workload, high pressure to perform, learning difficulties, poor time management, conflicts in work-life balance, conflictive personal relationships, inadequate peer relations, as well as health and financials problems (Hill et al., 2018). Cluster and factorial analyses led to identification of two groups with low and high stress, showing that women were more prone to stress than men. This is consistent with previous research showing higher distress in female students when exposed to human suffering (Hill et al., 2018), along with other findings pointing to gender differences in stress vulnerability, manifested through medical and psychiatric symptoms (Nechita et al., 2014).

Somatization resulting from of academic stress was higher in the group with high stress especially for drowsiness or increased need for sleep, anxiety, anguish, or desperation, chronic fatigue, and sleep disorders. An earlier study reported that early closure of academic institutions due to COVID-19 resulted in various psychological issues such as lack of sleep, need of emotional and mental support, and the search for social interactions (Nurunnabi et al., 2020).

Recent research on the psychological impact of the COVID-19 pandemic showed that individuals working in the clinical field had higher stress scores (Alkhamees et al., 2020), yet less was known about its impact among medical students. There is also evidence that female medical students had higher levels of stress, anxiety, and depression (Wang et al., 2020). Another study has shown that 71% of college students reported an increase in stress, anxiety levels, and depressive thoughts linked to the COVID-19 pandemic, which appeared to be associated with fear and concern with their own health and that of their loved ones; concentration difficulties, sleep disturbances, decrease in social interactions due to social distancing, and concern about their academic performance were also observed (Son et al., 2020).

In many settings, including the Mexican, medical students usually live in a high-stress environment, due in part to a rigid system that promotes competition. Thus, the COVID-19 pandemic seemed to aggravate this situation by adding new stress factors, including the concern and fear to the infection to oneself and relatives, and by limiting the benefits to engage in activities such as exercise and social interactions that would have reduced the stress; as a result, the lockdown and social distancing measures have led to radical lifestyle changes that took place in a very short period of time (Brooks et al., 2020).

This study found that the most common coping strategies included practicing a hobby, done always/nearly always by 65% of those with high stress and 34% of those with low stress, followed by getting company of a loved one (56 and 34%, respectively). Previous research on coping strategies among preclinical medical students highlight personal engagement, positive reinterpretation and expression of emotion, support programs delivered by senior students and extracurricular activities (mostly musical and physical activities) as valuable mechanisms to reduce anxiety, stress, and burnout (Fares et al., 2016). Other evidence has shown that medical students who adopt active coping, including planning and reality acceptance rather than avoiding strategies such as denial, alcohol and drug intake, and behavioral disengagement have better outcomes (Abouammoh et al., 2020). It has been seen that adaptive coping improves mental health while maladaptive coping strategies, such as negation, are predictors of depression among young adults (Mahmoud et al., 2012). Finally, another study with medical students from King Saud University also reported that mindfulness was associated with lower depressive symptoms (Alzahrani et al., 2020).

## 5. Conclusion

The results presented here reflect the impact of the prolonged COVID-19 pandemic in terms of the academic performance, occurrence of anxiety and depression, and stressors among medical students potentially linked to the lockdown accompanied with internet-based learning modality. Therefore, it is important to design and implement preventive strategies to deal with the effects of the online learning modality used during the COVID-19 lockdown.

### 5.1. Limitations

An important limitation relates to the observational design used which make it difficult to determine whether the COVID-19 pandemic could be indeed associated with the observed outcomes. Another limitation was the use of a non-probabilistic sample, and the fact that even in this sample selection bias is still a potential issue, as response rate went down to 76.1% for the Goldberg scale and to 44% for the SISCO-II inventory. Since reasons for not responding were not obtained, it was not possible to run a non-response analysis to compare participants and non-participants to speculate on the possible direction of the bias.

## Data availability statement

The original contributions presented in the study are included in the article/supplementary material, further inquiries can be directed to the corresponding authors.

## Ethics statement

Participation was voluntary. The questionnaire was administered *via* “Google forms.” Participants had to give informed consent in the first page of the questionnaire to be able to proceed with the survey. The study was conducted according to the guidelines of the Declaration of Helsinki and approved by the Institutional Review Board of Unidad Académica de Medicina Humana y C.S at Universidad Autónoma de Zacatecas (approval ID: AMC/CI/02-ORD/2020, CEICCL0121- ALGDEZA-03; approval date: November 6, 2020, and January 29, 2021).

## Author contributions

LA-C and MM-F conceptualization. LA-C and DD-A methodology. PV-E, SV-R, JS-G, and AM-G software. AR-L, JM-E, and IG-V validation. AR-L and JM-E formal analysis. AR-L and LA-C data curation and visualization. LA-C and DD-A writing—original draft preparation. JM-E, IG-V, JS-G, and MM-F writing—review and editing. All authors contributed to the article and approved the submitted version.

## Conflict of interest

The authors declare that the research was conducted in the absence of any commercial or financial relationships that could be construed as a potential conflict of interest.

## Publisher’s note

All claims expressed in this article are solely those of the authors and do not necessarily represent those of their affiliated organizations, or those of the publisher, the editors and the reviewers. Any product that may be evaluated in this article, or claim that may be made by its manufacturer, is not guaranteed or endorsed by the publisher.

## References

[ref1] Abdel Wahed W. Y. Hassan S. K. (2017). Prevalence and associated factors of stress, anxiety and depression among medical Fayoum university students. Alexandria J. Med. 53, 77–84. doi: 10.1016/j.ajme.2016.01.005

[ref2] Abouammoh N. Irfan F. Alfaris E. (2020). Stress coping strategies among medical students and trainees in Saudi Arabia: a qualitative study. BMC Med. Educ. 20:124. doi: 10.1186/s12909-020-02039-y, PMID: 32321498 PMC7178558

[ref3] Alkhamees A. A. Alrashed S. A. Alzunaydi A. A. Almohimeed A. S. Aljohani M. S. (2020). The psychological impact of COVID-19 pandemic on the general population of Saudi Arabia. Compr. Psychiatry 102:152192. doi: 10.1016/j.comppsych.2020.152192, PMID: 32688022 PMC7354380

[ref4] Alzahrani A. M. Hakami A. AlHadi A. Batais M. A. Alrasheed A. A. Almigbal T. H. (2020). The interplay between mindfulness, depression, stress and academic performance in medical students: a Saudi perspective. PLoS One 15:e0231088. doi: 10.1371/journal.pone.0231088, PMID: 32243468 PMC7122761

[ref5] Arribas Marín J. (2013). Hacia un modelo causal de las dimensiones del estrés académico en estudiantes de Enfermería. Rev. Educ., 533–556. doi: 10.4438/1988-592X-RE-2011-360-126

[ref6] Barraza A. (2007). Inventario SISCO del estrés académico. Propiedades psicométricas. Rev. Psicol. Cient. 9, 90–93.

[ref7] Barraza Macías A. (2008). Academic stress in master’s students and its modulatory variables: a between-groups design. Av. Psicol. Latinoam 26, 270–289.

[ref8] Behere S. P. Yadav R. Behere P. B. (2011). A comparative study of stress among students of medicine, engineering, and nursing. Indian J. Psychol. Med. 33, 145–148. doi: 10.4103/0253-7176.92064, PMID: 22345838 PMC3271488

[ref9] Blümel J. E. Fica J. Chedraui P. Mezones-Holguín E. Zuñiga M. C. Witis S.. (2016). Sedentary lifestyle in middle-aged women is associated with severe menopausal symptoms and obesity. Menopause 23, 488–493. doi: 10.1097/GME.0000000000000575, PMID: 26818013

[ref10] Brooks S. K. Webster R. K. Smith L. E. Woodland L. Wessely S. Greenberg N.. (2020). The psychological impact of quarantine and how to reduce it: rapid review of the evidence. Lancet 395, 912–920. doi: 10.1016/S0140-6736(20)30460-8 32112714 PMC7158942

[ref11] Brosschot J. F. Gerin W. Thayer J. F. (2006). The perseverative cognition hypothesis: a review of worry, prolonged stress-related physiological activation, and health. J. Psychosom. Res. 60, 113–124. doi: 10.1016/j.jpsychores.2005.06.074 16439263

[ref12] Castillo-Navarrete J. L. Guzmán-Castillo A. Claudio Bustos N. Walter Zavala S. Benjamín Vicente P. (2020). Psychometric properties of SISCO-II inventory of academic stress. Rev. Iberoam. Diagnost. Eval. Psicol. 56, 101–116. doi: 10.21865/RIDEP56.3.08

[ref13] Cohen S. Gianaros P. J. Manuck S. B. (2016). A stage model of stress and disease. Perspect. Psychol. Sci. 11, 456–463. doi: 10.1177/1745691616646305, PMID: 27474134 PMC5647867

[ref14] Crego A. Carrillo-Diaz M. Armfield J. M. Romero M. (2016). Stress and academic performance in dental students: the role of coping strategies and examination-related self-efficacy. J. Dent. Educ. 80, 165–172. doi: 10.1002/j.0022-0337.2016.80.2.tb06072.x, PMID: 26834134

[ref15] Dyrbye L. N. Harper W. Durning S. J. Moutier C. Thomas M. R. Massie F. S.. (2011). Patterns of distress in US medical students. Med. Teach. 33, 834–839. doi: 10.3109/0142159X.2010.531158 21942482

[ref16] Dyrbye L. N. Thomas M. R. Shanafelt T. D. (2005). Medical student distress: causes, consequences, and proposed solutions. Mayo Clin. Proc. 80, 1613–1622. doi: 10.4065/80.12.1613, PMID: 16342655

[ref17] Dyrbye L. N. Thomas M. R. Shanafelt T. D. (2006). Systematic review of depression, anxiety, and other indicators of psychological distress among U.S. and Canadian medical students. Acad. Med. 81, 354–373. doi: 10.1097/00001888-200604000-00009 16565188

[ref18] Eisenbarth H. Godinez D. du Pont A. Corley R. P. Stallings M. C. Rhee S. H. (2019). The influence of stressful life events, psychopathy, and their interaction on internalizing and externalizing psychopathology. Psychiatry Res. 272, 438–446. doi: 10.1016/j.psychres.2018.12.145, PMID: 30611961 PMC6428049

[ref19] Eva E. O. Islam M. Z. Mosaddek A. S. M. Rahman M. F. Rozario R. J. Iftekhar A. F. M. H.. (2015). Prevalence of stress among medical students: a comparative study between public and private medical schools in Bangladesh. BMC. Res. Notes 8:327. doi: 10.1186/s13104-015-1295-5, PMID: 26223786 PMC4520268

[ref20] Fares J. Al Tabosh H. Saadeddin Z. El Mouhayyar C. Aridi H. (2016). Stress, burnout and coping strategies in preclinical medical students. N. Am. J. Med. Sci. 8, 75–81. doi: 10.4103/1947-2714.177299 27042604 PMC4791902

[ref21] Fortuna G. Aria M. Cepeda-Valdes R. Garcia-Garcia S. C. Moreno Trevino M. G. Salas-Alanís J. C. (2016). Role of dystrophic epidermolysis bullosa in anxiety, depression and self-esteem: a controlled cross-sectional study. J. Dermatol. 43, 70–78. doi: 10.1111/1346-8138.13027, PMID: 26183725

[ref22] Goldberg D. Bridges K. Duncan-Jones P. Grayson D. (1988). Detecting anxiety and depression in general medical settings. Br. Med. J. 297, 897–899. doi: 10.1136/bmj.297.6653.897, PMID: 3140969 PMC1834427

[ref23] Gonzalez Heredia T. González-Ramírez L. P. Hernández-Corona D. M. Maciel-Hernández E. A. (2021). Anxious depression in patients with type 2 diabetes mellitus and its relationship with medication adherence and glycemic control. Glob. Public Health 16, 460–468. doi: 10.1080/17441692.2020.1810735, PMID: 32841093

[ref24] Guthrie E. A. Black D. Shaw C. M. Hamilton J. Creed F. H. Tomenson B. (1995). Embarking upon a medical career: psychological morbidity in first year medical students. Med. Educ. 29, 337–341. doi: 10.1111/j.1365-2923.1995.tb00022.x, PMID: 8699970

[ref25] Heinen I. Bullinger M. Kocalevent R. D. (2017). Perceived stress in first year medical students - associations with personal resources and emotional distress. BMC Med. Educ. 17, 4–14. doi: 10.1186/s12909-016-0841-8, PMID: 28056972 PMC5216588

[ref26] Hill M. R. Goicochea S. Merlo L. J. (2018). In their own words: stressors facing medical students in the millennial generation. Med. Educ. Online 23:1530558. doi: 10.1080/10872981.2018.1530558, PMID: 30286698 PMC6179084

[ref27] Holm J. Holm L. Bech P. (2001). Monitoring improvement using a patient-rated depression scale during treatment with anti-depressants in general practice: a validation study on the Goldberg depression scale. Scand. J. Prim. Health Care 19, 263–266. doi: 10.1080/02813430152706819, PMID: 11822653

[ref28] Hopkins Johns. (2020). “Coronavirus COVID-19 (2019-nCoV),”. Available at: https://gisanddata.maps.arcgis.com/apps/opsdashboard/index.html#/bda7594740fd40299423467b48e9ecf6 (Accessed July 04, 2021).

[ref29] Kalin N. H. (2020). The critical relationship between anxiety and depression. Am. J. Psychiatry 177, 365–367. doi: 10.1176/appi.ajp.2020.20030305 32354270

[ref30] Lasheras I. Gracia-García P. Lipnicki D. M. Bueno-Notivol J. López-Antón R. de la Cámara C.. (2020). Prevalence of anxiety in medical students during the covid-19 pandemic: a rapid systematic review with meta-analysis. Int. J. Environ. Res. Public Health 17, 1–12. doi: 10.3390/ijerph17186603 PMC7560147 32927871

[ref31] Loda T. Löffler T. Erschens R. Zipfel S. Herrmann-Werner A. (2020). Medical education in times of COVID-19: German students’ expectations – a cross-sectional study. PLoS One 15:e0241660. doi: 10.1371/journal.pone.0241660, PMID: 33206678 PMC7673791

[ref32] Mahmoud J. S. R. Staten R. T. Hall L. A. Lennie T. A. (2012). The relationship among young adult college students’ depression, an xiety, stress, demographics, life satisfaction, and coping styles. Issues Ment. Health Nurs. 33, 149–156. doi: 10.3109/01612840.2011.632708, PMID: 22364426

[ref33] Malo D. Cáceres G. Peña G. (2010). Validation of the inventory SISCO of the academic stress and comparative analysis in young adults from the Universidad Industrial de Santander UIS and the Universidad Pontificia Bolivariana, Sectional Bucaramanga. Prax. Invest. ReDIE 2, 26–42.

[ref34] Manrique-Millones D. Millones-Rivalles R. Manrique-Pino O. (2020). The SISCO inventory of academic stress: examination of its psychometric properties in a Peruvian sample. Ansiedad y Estrés 2019;25:28-34, and Chile JCastillo-NavarreteJL, Guzmán-Castillo a, Bustos-N C, Zavala-S W, Vicente-P B. psychometric properties of SISCO-II inventory of academic stress. Rev. Iberoam. Diagnóst. Eval. 56, 101–116. doi: 10.21865/RIDEP56.3.08

[ref35] Martín-Carbonell M. Pérez-Díaz R. Riquelme-Marín A. (2016). Diagnostic usefulness of anxiety and depression scale Goldberg (EAD-G) in Cuban adults. Univ Psychol 15, 177–192. doi: 10.11144/Javeriana.upsy15-1.vdea

[ref36] McEwen B. S. (1998). Protective and damaging effects of stress mediators. N. Engl. J. Med. 338, 171–179. doi: 10.1056/nejm199801153380307 9428819

[ref37] Moffat K. J. McConnachie A. Ross S. Morrison J. M. (2004). First year medical student stress and coping in a problem-based learning medical curriculum. Med. Educ. 38, 482–491. doi: 10.1046/j.1365-2929.2004.01814.x, PMID: 15107082

[ref39] Nechita F. Nechita D. Pîrlog M. C. Rogoveanu I. (2014). Stress in medical students. Romanian J. Morphol. Embryol. 55, 1263–1266. doi: 10.22037/jme.v11i1,2.1028 25607418

[ref40] Nelson B. W. Pettitt A. Flannery J. E. Allen N. B. (2020). Rapid assessment of psychological and epidemiological correlates of COVID-19 concern, financial strain, and health-related behavior change in a large online sample. PLoS One 15:e0241990. doi: 10.1371/journal.pone.0241990, PMID: 33175882 PMC7657530

[ref41] Nurunnabi M. Hossain S. F. A. H. Chinna K. Sundarasen S. Khoshaim H. B. Kamaludin K.. (2020). Coping strategies of students for anxiety during the COVID-19 pandemic in China: a cross-sectional study. F1000Research 9:1115. doi: 10.12688/f1000research.25557.1, PMID: 33274049 PMC7682494

[ref42] Pozos-Radillo E. Preciado-Serrano L. Plascencia-Campos A. Valdez-López R. Morales-Fernández A. (2016). Psychophysiological manifestations associated with stress in students of a public University in Mexico. J. Child Adolesc. Psychiatr. Nurs. 29, 79–84. doi: 10.1111/jcap.12142, PMID: 27279437

[ref43] Puthran R. Zhang M. W. B. Tam W. W. Ho R. C. (2016). Prevalence of depression amongst medical students: a meta-analysis. Med. Educ. 50, 456–468. doi: 10.1111/medu.12962, PMID: 26995484

[ref44] Putwain D. (2007). Researching academic stress and anxiety in students: some methodological considerations. Br. Educ. Res. J. 33, 207–219. doi: 10.1080/01411920701208258

[ref45] Reivan-Ortiz G. Pineda-Garcia G. León-Parias B. D. (2019). Psychometric properties of the Goldberg anxiety and depression scale (GADS) in Ecuadorian population. Int J Psychol Res 12, 41–48. doi: 10.21500/20112084.3745, PMID: 32612786 PMC7110168

[ref46] Segerstrom S. C. Schipper L. J. Greenberg R. N. (2008). Caregiving, repetitive thought, and immune response to vaccination in older adults. Brain Behav. Immun. 22, 744–752. doi: 10.1016/j.bbi.2007.11.004, PMID: 18166335 PMC2464708

[ref47] Selye H. (1948). The alarm reaction and the diseases of adaptation. Ann. Intern. Med. 29, 403–415. doi: 10.7326/0003-4819-29-3-403, PMID: 18882927

[ref48] Son C. Hegde S. Smith A. Wang X. Sasangohar F. (2020). Effects of COVID-19 on college students’ mental health in the United States: interview survey study. J. Med. Internet Res. 22:e21279. doi: 10.2196/21279 32805704 PMC7473764

[ref49] Straub R. H. Cutolo M. (2018). Psychoneuroimmunology—developments in stress research. Wien. Med. Wochenschr. 168, 76–84. doi: 10.1007/s10354-017-0574-2 28600777

[ref50] Tafet G. E. Nemeroff C. B. (2016). The links between stress and depression: Psychoneuroendocrinological, genetic, and environmental interactions. J. Neuropsychiatry Clin. Neurosci. 28, 77–88. doi: 10.1176/appi.neuropsych.15030053, PMID: 26548654

[ref51] Toews J. A. Lockyer J. M. Dobson D. J. Simpson E. Brownell A. K. Brenneis F.. (1997). Analysis of stress levels among medical students, residents, and graduate students at four Canadian schools of medicine. Acad. Med. 72, 997–1002. doi: 10.1097/00001888-199711000-00019, PMID: 9387825

[ref52] Ursin H. Eriksen H. R. (2010). Cognitive activation theory of stress (CATS). Neurosci. Biobehav. Rev. 34, 877–881. doi: 10.1016/j.neubiorev.2009.03.001, PMID: 20359586

[ref53] Wang C. Pan R. Wan X. Tan Y. Xu L. Ho C. S.. (2020). Immediate psychological responses and associated factors during the initial stage of the 2019 coronavirus disease (COVID-19) epidemic among the general population in China. Int. J. Environ. Res. Public Health 17:1729. doi: 10.3390/ijerph17051729, PMID: 32155789 PMC7084952

[ref54] World Health Organization. (2022). “Coronavirus disease (COVID-19),”. Available at: https://www.who.int/emergencies/diseases/novel-coronavirus-2019 (Accessed December 05, 2022).

[ref55] Yang P. Wang X. (2020). COVID-19: a new challenge for human beings. Cell. Mol. Immunol. 17, 555–557. doi: 10.1038/s41423-020-0407-x 32235915 PMC7110263

[ref56] Zeppa B. S. Linn R. (1984). Stress in junior medical students: relationship to personality and performance. J. Med. Educ. 59, 7–12. 6690704

[ref57] Zhai Y. Du X. (2020). Addressing collegiate mental health amid COVID-19 pandemic. Psychiatry Res. 288:113003. doi: 10.1016/j.psychres.2020.113003 32315885 PMC7162776

[ref58] Zvauya R. Oyebode F. Day E. J. Thomas C. P. Jones L. A. (2017). A comparison of stress levels, coping styles and psychological morbidity between graduate-entry and traditional undergraduate medical students during the first 2 years at a UK medical school. BMC. Res. Notes 10:93. doi: 10.1186/s13104-017-2395-1, PMID: 28193287 PMC5307866

